# The feasibility and positive effects of a customised videogame rehabilitation programme for freezing of gait and falls in Parkinson’s disease patients: a pilot study

**DOI:** 10.1186/s12984-018-0375-x

**Published:** 2018-04-10

**Authors:** Dijana Nuic, Maria Vinti, Carine Karachi, Pierre Foulon, Angèle Van Hamme, Marie-Laure Welter

**Affiliations:** 1 CNRS, UMR7225, Institut du Cerveau et de la Moelle Epinière, Sorbonne universités, Université Pierre et Marie Curie (UPMC) Paris P6; UMRS 1127, 75013 Paris, France; 2 LabCom BRAIN e-NOVATION, Institut du Cerveau et de la Moelle épinière (ICM), 75013 Paris, France; 3 Neurosurgery Department, Hôpital de la Salpêtrière, Groupe Hospitalier Pitié-Salpêtrière, APHP, 75013 Paris, France; 4 GENIOUS System, 92700 Colombes, France; 5 PANAM Platform, Institut du Cerveau et de la Moelle épinière, 75013 Paris, France; 6 Neurophysiology Department, Rouen University Hospital, Rouen-Normandie University, 76000 Rouen, France

**Keywords:** Parkinson’s disease, Freezing of gait, Falls, Videogame, Rehabilitation

## Abstract

**Background:**

Freezing of gait and falls represent a major burden in patients with advanced forms of Parkinson’s disease (PD). These axial motor signs are not fully alleviated by drug treatment or deep-brain stimulation. Recently, virtual reality has emerged as a rehabilitation option for these patients. In this pilot study, we aim to determine the feasibility and acceptability of rehabilitation with a customised videogame to treat gait and balance disorders in PD patients, and assess its effects on these disabling motor signs.

**Methods:**

We developed a customised videogame displayed on a screen using the Kinect system. To play, the patient had to perform large amplitude and fast movements of all four limbs, pelvis and trunk, in response to visual and auditory cueing, to displace an avatar to collect coins and avoid obstacles to gain points. We tested ten patients with advanced forms of PD (median disease duration = 16.5 years) suffering from freezing of gait and/or falls (Hoehn&Yahr score ≥ 3) resistant to antiparkinsonian treatment and deep brain stimulation. Patients performed 18 training sessions during a 6–9 week period. We measured the feasibility and acceptability of our rehabilitation programme and its effects on parkinsonian disability, gait and balance disorders (with clinical scales and kinematics recordings), positive and negative affects, and quality of life, after the 9th and 18th training sessions and 3 months later.

**Results:**

All patients completed the 18 training sessions with high feasibility, acceptability and satisfaction scores. After training, the freezing-of-gait questionnaire, gait-and-balance scale and axial score significantly decreased by 39, 38 and 41%, respectively, and the activity-balance confidence scale increased by 35%. Kinematic gait parameters also significantly improved with increased step length and gait velocity and decreased double-stance time. Three months after the final session, no significant change persisted except decreased axial score and increased step length and velocity.

**Conclusions:**

This study suggests that rehabilitation with a customised videogame to treat gait and balance disorders is feasible, well accepted, and effective in parkinsonian patients. These data serve as preliminary evidence for further larger and controlled studies to propose this customised videogame rehabilitation programme at home.

**Trial registration:**

ClinicalTrials.gov NCT02469350.

## Background

Parkinson’s disease (PD) is the second most common neurodegenerative disorder resulting mainly from the loss of dopaminergic midbrain neurons. Clinically, this disease is characterised by akinesia, with motor slowness, decreased amplitude of automatic movements and movement initiation failure, rigidity and tremor [1], and is improved by dopamine replacement therapy [2]. Patients with PD also present non-motor signs with dysexecutive syndrome, apathy, anxiety and depression [3]. Up to now, no treatment exists to arrest or slow down the neurodegenerative process. In advanced and severe forms, freezing of gait (FOG) and postural instability with falls occur in about 50–75% of patients [4]. These signs worsen with time and become unresponsive to drug treatment or deep-brain stimulation [5], leading to increased mortality and morbidity, poor life quality and high healthcare costs [6].

In PD patients, physiotherapy represents an additional treatment to maximise functionality through movement rehabilitation. It focuses on transfers, posture, upper limb function, balance, gait, and physical activity, also including cueing strategies, cognitive movement strategies, and exercise [7]. Various techniques have been proposed within physiotherapy with general physiotherapy, exercise, treadmill walking, cueing (with auditory, visual or somatosensory cues), dance or martial arts, with short-term improvements of gait speed, freezing of gait, balance and falls, global motor function, activities of daily living, and quality of life [7], with no clear evidence of differences in the short-term effects of treatment between physiotherapy methods [7, 8], but a better outcome if balance dysfunction is addressed [8]. A recent meta-analysis highlights the long-term positive effects of physical therapy with improved gait and balance performance, enhanced activities of daily living and quality of life, in particular for progressive resistance training, as long as it is maintained over time [9]. Finally, the majority of studies addressing the effects of physiotherapy and exercise have revealed a loss of benefit within weeks or months after discontinuation of training [8]. Moreover, several barriers for exercise compliance have been identified in people with PD with low perceived benefit of exercise, lack of time, as well as fear of falling [10]. The use of virtual reality has therefore emerged as a promising rehabilitation option for PD with the possibility of improving long-term adherence to exercise in a personalised, motivating and engaging manner [11]. Recent studies have suggested that virtual reality training, such as combined with treadmill walking [12, 13] or cycling [14], produces short-term benefits on gait, balance, activities of daily living, cognition, quality of life, with good exercise adherence and no adverse events [15]. Treadmill walking coupled with virtual reality also promotes fall reduction 6 months after the cessation of training [13]. Virtual reality exercises stimulate movement, optimise motor learning, potentially compensate defective internal mechanisms using external cueing and allow augmented feedback [14]. In addition to a challenging and motivating virtual environment, gaming also allows training for specific motor and non-motor signs with an amusing and enjoyable approach, that can be performed at home with increased training frequency and/or on demand [16]. A few studies have tested the effects of commercial games using Nintendo Wii™ or Xbox Kinect™ systems in PD patients and reported good feasibility [17] with increased balance [18–20] and activities of daily living [21, 22] performance, mood and quality of life [22]. In some of these previous studies, the effects of commercial games were not specific to the disease with similar changes in healthy controls after training [18, 23]. These PD patients also only suffered from mild to moderate forms of PD, with no disabling or refractory gait and balance disorders. Lastly, training with commercial games may not be suitable for such PD patients due to their specific motor disability [17]. One customised game has recently been tested for arm movement training in 7 PD patients with encouraging results [24].

Here, we propose a virtual reality training system using a customised game specifically designed to treat refractory gait and balance disorders of PD patients according to recommended physiotherapy programmes for PD patients with high amplitude and fast movements, combined with visual and auditory cueing [25]. The aim of this pilot study was to assess the feasibility and acceptability of our customised videogame in 10 PD patients with FOG and/or postural instability with falls resistant to dopaminergic treatment and deep brain stimulation. We examined the effects of our game training on gait and balance control by using both clinical and kinematic gait recordings, with a final examination of the patients 3 months after the last session in order to assess the persistence of training benefits.

## Methods

### Study design

This study was a pilot study. Eligible participants consisted of PD patients. This study is part of clinical trial C15–12 sponsored by Inserm (ID RCB: 2015-A00277–42). It was granted approval by local Ethics Committee (Comité de protection des personnes-Ile de France V on June 2, 2015), authorised by the French authorities (ANSM, 150358B-31), and registered in a public trials registry (Trial Registration: ClinicalTrials.gov NCT02469350). All study participants gave their informed, written consent to participation, in line with French ethical guidelines.

### The ‘Toap run’ videogame

The videogame (‘Toap Run’) was built specifically for this study. The videogame was controlled by a laptop (Asus®, Model G551 J, Intel® Core™ i7, NVIDIA® GEFORCE® GTX TM8, 8 GB of RAM) and displayed on a high definition television screen (Phillips, 50-in. plasma, resolution 1920 by 1080 pixels) placed 2 m in front of the patient. The patient played by moving in front of a RGB-D (Red Green Blue + Depth) Kinect™ motion sensor (Version 2, Microsoft, USA) placed below the screen.

**Fig. 1:**
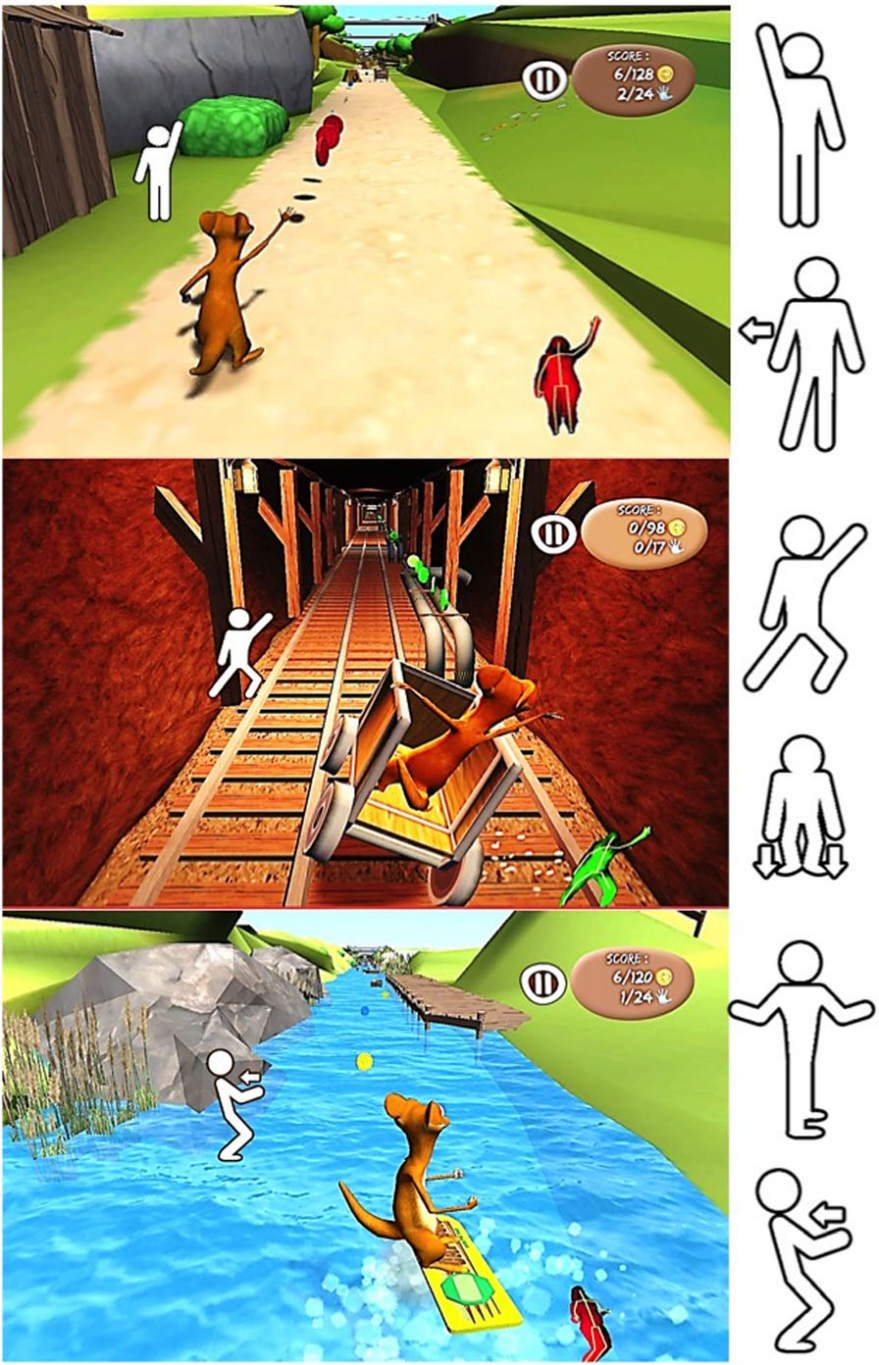
The ‘Toap Run’ videogame training. The images show screen shots of one patient during videogame training with ‘Toap Run’ in the three different scenarios. From Top to Bottom: ‘The Garden’, ‘The Mine’, and ‘The River’. The movements are schematically represented to the side of the images, from top to bottom: arm extension, lateral shift, trunk lateral displacement with knee flexion, knee flexion/extension, trunk rotation with arm movements, and anteroposterior trunk movement

The scenario consists of a small animal (the avatar) that moves around three different environments: a garden, a mine cart and a surfboard on a lake (Fig. 1). To play, the patient, standing upright in front of the screen, has to move to induce displacements of the avatar in real time within these different environments, to collect coins and avoid obstacles to gain points, in relationship to visual and auditory cues. The movements consist of large amplitude rapid movements of all four limbs, pelvis and trunk, with lateral, vertical and forward displacements of the legs, to reinforce foot lifting and postural control (Fig. 1):

- In ‘The Garden’, the patients had to extend their arms to collect coins and perform lateral displacements to avoid laterally placed obstacles,
- In ‘The Mine’, the patients had to collect coins and avoid obstacles with the same movements as in ‘The Garden’, plus knee flexion/extension to avoid high level obstacles and lateral trunk displacements with one leg flexion to move the wagon and collect coins,
- In ‘The River’, the patients had to perform trunk rotation and arm movements to collect coins, and anteroposterior trunk movements to avoid obstacles.

Visual cueing consisted of a schematic representation of the movements required to perform along the trajectory of the avatar. Auditory cueing consisted of rhythmic music that beats in relationship to the velocity of the avatar displacement. Three different rhythms were presented: 20, 30 and 40 beats/min. The first session was stereotyped and consisted of a 15 min session with a 20 beat velocity first in the garden and then in the mine, corresponding to a total of 100–150 movements. Game difficulty, i.e. velocity and number of movements to capture the coins and avoid obstacles, was then adjusted manually and individually by the physiotherapist for subsequent rehabilitation sessions, in relation to the patient’s ability. For this purpose, the physiotherapist was instructed to increase the rhythm, number and complexity of movements, i.e. the environment, depending on the performance in the previous session with a view to obtain a session of at least 40 min duration after the 9th session [26].

### Participants

Between June 2015 and May 2016, ten people with PD (mean age: 64.2 ± 6.1 years, 5F/5M) were recruited at the Pitié-Salpêtrière Hospital and included according to the following criteria: 1) diagnosis of PD, 2) age below 71 years, 3) disabling FOG and/or falls despite optimal drug treatment and subthalamic deep-brain stimulation (items 13-falls and/or item 14-FOG of the UPDRS part II ≥ 2 ON treatment) [27] (Table 1). Exclusion criteria included dementia (Mini Mental State Examination Score < 24), inability to maintain upright posture or walk independently, patients suffering from other pathologies affecting gait and balance, or severe medical conditions that prevented assessment.

**Table 1:** Clinical and demographic features of PD patients at inclusion

N° patient	Sex/Age (yrs)	Disease duration (yrs)	Time since STN-DBS (months)	Falls/FOG duration (months)	UPDRS part II: falls/FOG	UPDRS part III (/108)	Hoehn&Yahr stage (/5)	FOG-Q (/64)	ABC scale (%)	LEED (mg/day)
01	M/58	13	26	20	2/3	21	3	32	70	700
02	F/68	21	52	14	3/2	27	4	32	39	700
03	M/62	15	60	30	2/3	20	3	22	51	350
04	F/69	16	93	80	2/2	13	3	27	46	300
05	M/70	17	18	13	2/3	15	3	21	69	600
06	M/69	32	183	159	4/4	32	4	30	50	200
07	F/69	20	34	26	2/3	13	3	20	22	600
08	F/61	13	37	25	2/2	14	3	17	64	400
09	F/53	23	140	65	2/3	15	3	30	31	300
10	M/61	11	20	18	2/3	33	4	28	72	700
Mean (SD)	64.0 (5.8)	18.1 (6.2)	66.3 (55.8)	45.0 (45.9)	2.3 (0.7)/2.8 (0.6)	20.3 (7.8)	3.3 (0.5)	25.9 (5.4)	51.4 (17.3)	485 (205)

*ABC* activity balance confidence, *FOG-Q* freezing of gait questionnaire, *LEED* levodopa-equivalent dosage, *STN-DBS* subthalamic deep brain stimulation, *UPDRS* unified Parkinson’s disease rating scale

### Procedure

The videogame rehabilitation programme consisted of 18 training sessions with the ‘Toap Run’ videogame over a period of 6 weeks, in addition to the usual anti-parkinsonian treatment, which was unchanged throughout the duration of the protocol. Training was performed in the institute and supervised by a physiotherapist (DN).

Clinical assessments and kinematic parameters of gait and balance disorder recordings were performed at inclusion (before the first training session), after the 9th and 18th training sessions, and 3 months later.

#### Gait initiation recordings

The gait initiation process was specifically studied as this requires simultaneous forward movement (locomotion) and balance control, in order to maintain stability and prevent falling [28]. Kinematic parameters of gait initiation were recorded using a force platform (0.9 × 1.8 m, AMT Inc. LG6–4-1). Subjects, barefoot and standing upright, were instructed to commence walking for 5 m following an auditory cue. Ten consecutive trials were recorded, the subject being instructed to walk at a self-paced speed [29].

### Outcome measures

The primary outcome measure was the feasibility of the videogame rehabilitation programme using a Likert-scale (1 to 7 points per item) specific questionnaire [30]. With this scale we measured the perceived interest (4 items, with scores ranging from 4 to 28, higher scores indicating better perceived interest), perceived competence (8 items, with scores ranging from 8 to 56, higher scores indicating higher perceived competence) and perceived difficulty (3 items, with scores ranging from 3 to 21, lower scores indicating lower perceived difficulty). This questionnaire was performed after the first training and once a week.

Secondary outcomes included the acceptability scale evaluating the level of interest, satisfaction and discomfort in the game by using a visual analogue scale ranging from zero to 10. Perceived fatigue was evaluated by using the ‘sensory’ and ‘cognition’ subscales of the Piper Fatigue Revised Scale (with scores ranging from 0 to 10, higher scores indicating higher perceived fatigue) [31], and perceived affects using the Positive and Negative Emotionality questionnaire (EPN-31) that comprised 31 items quantifying the frequency of each affect (7-point scale, ranging from 1 “not experienced at all” to 7 “experienced this affect several times each day”) with 3 subscores: positive, negative and surprise affects [32]. The users’ experience in the game was assessed using a 5-point Likert scale. Game performance (percentage of coins captured), number of movements and duration of each training session were also recorded. These questionnaires were administered after the first session and once a week.

Other secondary outcomes focused on gait and balance disorders including the parkinsonian motor disability (UPDRS part III, range = 0 to 108) [27], ‘axial’ (sum of items 28–29–30-31 of the UPDRS part III, range = 0 to 16), gait and balance scale (GABS part B- clinical examination, range = 0 to 38) [33], FOG questionnaire (FOG-Q, range = 0 to 64) [34], fear of falling (Activity Balance Confidence-ABC, range = 0 to 100%) [35], activities of daily living (ADL-UPDRS part II, range = 0 to 42) [27] and quality of life (Summary index of the Parkinson’s disease Questionnaire-39: SI-PDQ39, range = 0–100%) [36] scales. Higher scores in these scales indicate greater disability and/or more severe signs, except the ABC scale for which higher scores indicate higher confidence. The fall frequency was assessed with item 12 of the FOG-Q: *‘How often do you fall?’* with scores ranging from 0 to 4 (0 = Never, 1 = Very rarely-about once a year, 2 = rarely-about once a month, 3 = often- about once a week, 4 = very often-once a day or more) [34].

**Fig. 2:**
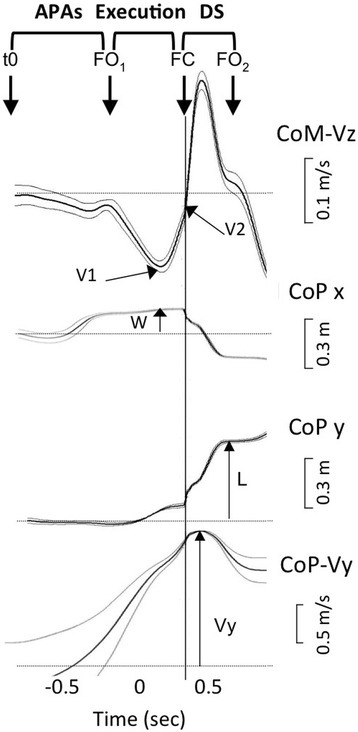
Kinematic parameter recordings of gait initiation. Kinematic parameters of gait initiation in an individual patient. From top to bottom, curves represent the smoothed mean of ten trials and show the vertical centre of mass velocity (CoM-Vz), mediolateral (CoP x) and anteroposterior (CoP y) displacements and anteroposterior centre of foot pressure (CoP) velocity (CoP-Vy). APAs: anticipatory postural adjustments, DS: double-stance, CoP: centre of foot pressure, CoM: centre of mass, FC: foot contact of the swing leg, FO1: foot-off of the swing leg, FO2: foot-off of the stance leg, L: step length, t0: time of the first biomechanical event, V1: negative peak of the CoM vertical velocity, V2: CoM vertical velocity at the time of foot contact, W: step width

Gait initiation kinematics parameters were the anteroposterior and lateral displacements of the centre of foot pressure (CoP) during the anticipatory postural adjustments (APAs) phase, length and velocity of the first step and braking index (reflecting active postural control) [29, 37] (Fig. 2). The duration of the APAs phase (time between the first biomechanical event and foot-off of the swing foot) and double stance time (time between the foot-contact of the swing foot and foot-off of the stance foot) were also measured (Fig. 2). Clinical assessments and gait recordings were performed at baseline (inclusion), after the 9th and 18th sessions, and 3 months later, in the morning with usual antiparkinsonian treatment and stimulation parameters.

### Statistical analysis

To assess the changes in the feasibility, acceptability, perceived fatigue, emotion questionnaires, motivation, movements during gaming and game duration, we first tested the data distribution using the Shapiro-Wilk test and the variance homogeneity using Levene’s test. If data were found to be normally distributed and show homogeneity, we performed repeated measures ANOVAs, and in case of non-normal distribution, non-parametric Friedman’s ANOVAs. If the results were significant, Tukey’s post-hoc tests were performed for parametric data, and Nemenyi post-hoc tests for Friedman’s ANOVA.

The changes in the clinical scores between baseline and after the 9th and 18th sessions, and 3 months later were assessed using the non-parametric Wilcoxon tests. We used the MacNemar test to measure the change in fall frequency between baseline and after the 18th session.

Lastly, the changes in the kinematic gait parameters were measured using a linear mixed model.

All statistical analyses were performed using the R package lme4 (version 1.1–12, R-studio Inc., R Core Development Team). Results were considered significant at *p* < 0.05.

## Results

### Game training sessions

**Fig. 3:**
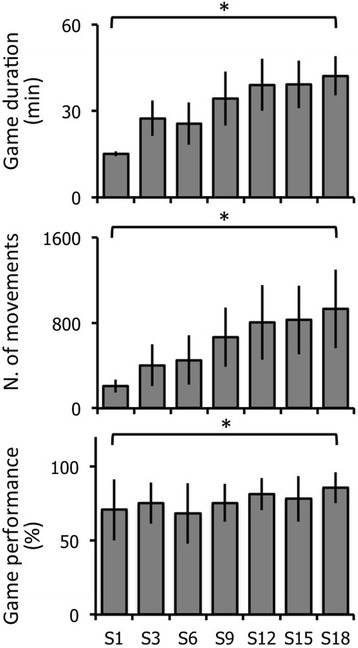
Game duration, difficulty and performance in PD patients. The graphs represent the mean and standard deviation, from top to bottom, in game duration, difficulty (number of movements) and performance after the 1st (S1), 3rd (S3), 6th (S6), 9th (S9), 12th (S12), 15th (S15) and 18th (S18) training sessions. * *p* < 0.05 repeated measures ANOVAs

All patients performed the 18 training sessions. Game duration, game difficulty (with increased visual flow of the virtual environment and number of movements to perform) and game performance increased significantly over time (Friedman’s ANOVA; Game duration: 34.0 ± 10.3 min, χ2 [17] =102.3, *p* < 10^− 4^; Number of movements: 654.2 ± 351.9, χ2 [17] =120.9, *p* < 10^− 4^; Game performance: 76.3% ± 14.5%, χ2 [17] = 32.3, *p* = 0.01, Fig. 3, Table 2). Post-hoc analysis revealed that game duration increased significantly between the first and 8th session and those following, the 2nd and 10th session and those following, the 3rd and the 12th session and those following and between the 4th, 5th and 6th sessions and the 16th to the 18th session (Table 2). Similarly, the number of movements significantly increased between both the 1st and 2nd sessions and the 10th and those following, and between the 3rd, 4th, 5th and 6th sessions and the 14th to the 18th session (Table 2). Lastly, post-hoc analysis revealed that the game performance had increased significantly between the 6th and 18th sessions (Table 2).

**Table 2:** Number of movements, game duration and performance of PD patients over training sessions

N° session	Game duration (min)	Number of movements	Game performance (%)
01	15.1 (0.8)	207 (61)	70.8 (20.8)
02	22.0 (6.2)	323 (120)	72.6 (15.9)
03	27.5 (6.2)	403 (195)	75.3 (13.9)
04	28.9 (2.9)	448 (165)	75.6 (16.6)
05	26.7 (6.1)	440 (246)	71.5 (17.6)
06	25.7 (7.4)	452 (230)	68.4 (20.6)
07	33.8 (4.6)	615 (270)	79.5 (12.6)
08	36.7 (5.5)^a^	626 (154)	76.4 (13.4)
09	34.4 (9.4)^a^	666 (280)	75.4 (12.9)
10	40.4 (10.2)^a,b^	813 (380)^a,b^	74.3 (9.1)
11	38.5 (7.1)^a,b^	785 (241)^a,b^	77.0 (12.6)
12	39.1 (9.1)^a,b,c,^	806 (351)^a,b^	75.0 (18.1)
13	37.9 (6.0)^a,^	744 (285)^a,b^	81.5 (10.9)
14	39.8 (4.4)^a,b,c,f^	860 (354)^a,b,c,d,e,f^	76.0 (11.2)
15	39.2 (8.3)^a,b,c^	827 (322)^a,b,c,d,ef^	79.5 (12.8)
16	41.6 (12.1)^a,b,c,d,e,f^	899 (438)^a,b,c,d,e,f^	78.2 (15.4)
17	42.9 (7.6)^a,b,c,d,e,f^	931 (410)^a,b,c,d,e,f^	81.9 (11.9)
18	42.2 (6.8)^a,b,c,d,e,f^	932 (368)^a,b,c,d,e,f^	85.8 (10.5)^f^

Values are mean (SD)

^a^*p* < 0.05 as compared to Session 01

^b^*p* < 0.05 as compared to Session 02

^c^*p* < 0.05 as compared to Session 03

^d^*p* < 0.05 as compared to Session 04

^e^*p* < 0.05 as compared to Session 05

^f^*p* < 0.05 as compared to Session 06

### Feasibility, acceptability and emotional perception

**Fig. 4:**
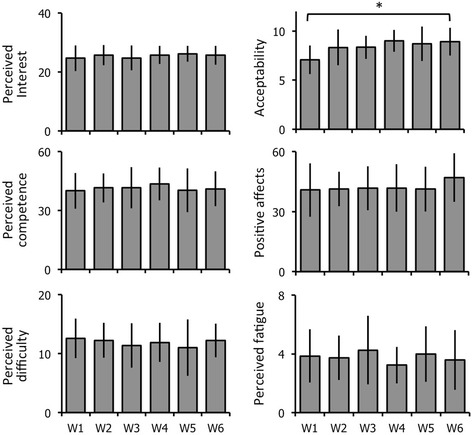
Acceptability and feasibility of the ‘Toap Run’ videogame training in PD patients. The graphs represent the mean and standard deviation, from left to right and top to bottom, in perceived interest, perceived competence, perceived difficulty, perceived fatigue, acceptability and positive affects after the 1st session (W1) and once a week from the 2nd to the 6th week (W2 to W6). * *p* < 0.05 repeated measures ANOVAs

Patients reported high-perceived interest and competence scores for videogame training, these scores were stable over time (Friedman’s ANOVA; Interest: 25.4 ± 3.4; χ^2^ [5] =10.6, *p* = 0.06; ANOVA; competence: 41.3 ± 9.1; F [5, 45] = 0.92, *p* = 0.47, Fig. 4). Perceived difficulty was low and also stable over time (ANOVA, 11.85 ± 3.4; F [5, 45] = 0.36, *p* = 0.87, Fig. 4). General acceptability increased significantly over time (Friedman’s ANOVA, 8.39 ± 1.56; χ^2^ [5] = 20.5, *p* = 10^− 4^, Fig. 4). Post-hoc analysis revealed that general acceptability increased between the 1st and 4th week and was maintained at high levels after. Perceived fatigue and negative affects were variable among patients with no significant change over time (Friedman’s ANOVA; Fatigue: 3.77 ± 1.79; χ^2^ [5] = 3.893, *p* = 0.56; Negative affects: 42.85 ± 16.66; χ^2^ [5] = 3.084, *p* = 0.69, Fig. 4). Positive affects tended to decrease over time (ANOVA, 42.3 ± 11.2; F [5, 45] = 1.879, *p* = 0.11)*.* Lastly, 7 out of 10 patients rated the game as amusing.

### Clinical gait and balance disorders, parkinsonian disability and quality of life

**Fig. 5:**
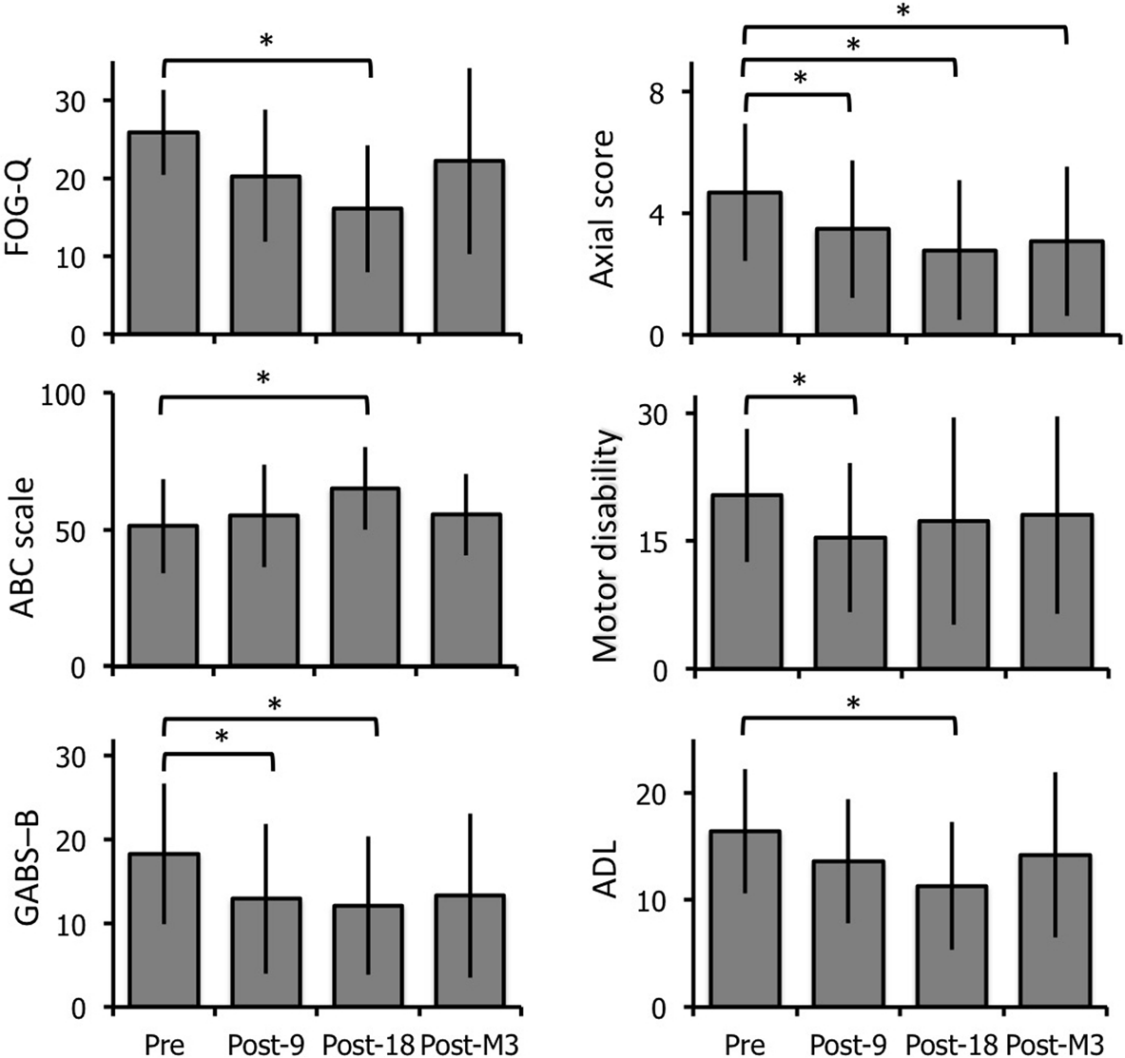
Effects of the ‘Toap Run’ videogame training on gait and balance disorders, parkinsonian disability and quality of life in PD patients. The graphs represent the mean and standard deviation, from left to right and top to bottom, in the Freezing Of Gait Questionnaire (FOG-Q), Activities and Balance Confidence (ABC) scale, Gait And Balance scale part B (GABS-B), Axial score (UPDRS items 18 + 27 + 28 + 29 + 30), parkinsonian motor disability (UPDRS part III) and activities of daily living (ADL, UPDRS part II) scores. Results were obtained before the first (Pre), after the 9th (Post-9) and 18th (Post-18) training sessions, and 3 months later (Post-M3). * *p* < 0.05 as compared to the first (baseline) assessment

After the 18th session, when compared to baseline, the FOG-Q, GABS-B, and axial scores significantly decreased by 39, 38 and 41%, respectively (*p* = 0.005, 0.02 and 0.009, respectively, Fig. 5) and ABC scale significantly increased by 35% (*p* = 0.03, Fig. 5). The GABS-B and axial scores also significantly decreased by 36 and 15%, respectively, after the 9th session (*p* = 0.04 and *p* = 0.008, respectively, Fig. 5). All patients presented recurrent falls at baseline (FOG-Q-item 12 > 1). After the 18th session, seven of the 10 patients reported no falls (*p* = 0.023, not shown).

Parkinsonian motor disability (UPDRS part III, Fig. 5) and quality of life (PDQ-39, not shown) showed no significant change after the 18th training session relative to baseline (*p* = 0.34 and 0.13, respectively), but the parkinsonian motor disability was significantly lower after the 9th session (*p* = 0.02, Fig. 5). The ‘Activities of daily living’ (UPDRS part II) score significantly decreased after the 18th session in comparison to baseline (*p* = 0.03, Fig. 5).

Three months later, we found no changes in the clinical scores, except a persistent significant decrease of 35% in the axial score compared to baseline (*p* = 0.005, Fig. 5), and eight of ten patients reported recurrent falls again.

### Gait initiation recordings

**Fig. 6:**
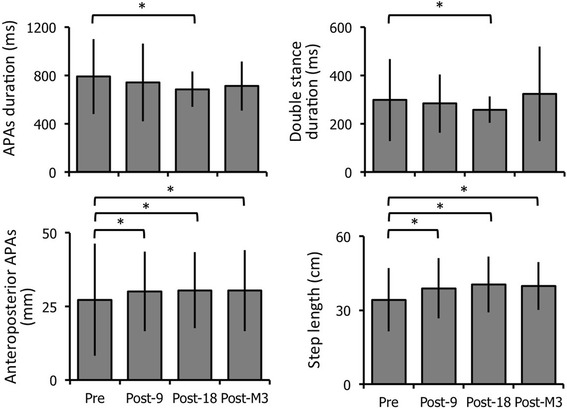
Effects of the ‘Toap Run’ videogame training on gait initiation kinematic parameters. Each graph represents the mean and standard deviation from left to right and top to bottom of the APAs phase and double-stance durations, and anteroposterior CoP displacements during APAs and step length. Results were obtained before (Pre), after the 9th (Post-9) and 18th (Post-18) sessions, and 3 months later (Post-M3). * *p* < 0.05 as compared to the first (Pre) assessment

After the 18th session, the APAs phase and double-stance durations were significantly lower (*p* = 0.0004 and 0.002, respectively, Fig. 6) compared to baseline, and the anteroposterior APAs displacement, step length (*p* < 10^− 4^, Fig. 6) and gait velocity (*p* < 10^− 4^, not shown) significantly increased. The mediolateral APAs displacement, step width and braking index showed no significant change (not shown). The anteroposterior APAs, step length and velocity were also significantly higher after the 9th session and 3 months later, compared to baseline (Fig. 6).

### Adverse events

Three serious adverse events, unrelated to the training, were reported in three patients. Severe pneumonia occurred in one patient (P02) and a deep brain stimulation generator replacement was required in two patients (P06 and P09). The videogame training sessions were well tolerated with no specific adverse events over the course of the study.

## Discussion

The primary aim of this pilot study was to develop and evaluate the feasibility of a videogame rehabilitation programme using a specific customised game that targeted FOG and falls in persons with advanced and severe forms of PD. The results indicate that our videogame is feasible for use in this patient population with good overall satisfaction and motivation to use the game for training, increased performance and duration of gaming with time. All the patients gave a positive opinion about the videogame and the virtual environments proposed. Clinical assessment revealed focussed improvement in gait and balance disorders with significantly decreased severity in FOG and reduction of falls, that could be transferred to increased activities of daily living competence with better confidence in balance and increased positive affects. These clinical effects were confirmed by objective kinematic recordings showing increased anteroposterior APAs displacement, step length and velocity, which reflect better gait performance, and decreased double stance and APAs duration, which reflect better balance control [29, 38]. The majority of these positive effects were not maintained, however, 3 months later.

This study has some limitations. These results were obtained in a small group of patients with no control group or comparative treatment, with many secondary outcomes at different time points, thus limiting the generalisation of our findings. However, from an individual point of view, all patients reported good feasibility and acceptability, with decreased severity of gait and balance disorders after training. The training was performed at the institute in the presence of the physiotherapist. This may have increased the acceptability and motivation of the patients to perform the training programme. However, 6 out of 10 patients asked to perform gaming at home at the end of the programme suggesting that our game was indeed well accepted and perceived as feasible by our patients, even in the absence of the physiotherapist. Future research with game training at home in the absence of the physiotherapist could help to decipher the respective role of the customised videogame and of the physiotherapist supervision in the effects of this rehabilitation programme. We did not specifically examine the effects of our videogame rehabilitation programme on cognition, knowing that PD patients classically presented dysexecutive syndrome. In our study, the number of secondary outcomes with patient’s interviews limited the possibility to add complete neuropsychological testing. This should be performed in future studies.

The use of commercial game training for PD rehabilitation has previously been reported in small groups of patients suffering mild to moderate forms of PD (Hoehn and Yahr score stages ranging from 1 to 3, disease duration generally less than 10 years), with good feasibility, mild improvement in parkinsonian disability, increased single-leg stance duration, dynamic balance and gait velocity, activities of daily living, quality of life and cognition [15]. Here, we tested our videogame training in severe and advanced forms of PD with resistant gait and balance disorders. These results indicate that our intervention which required high amplitude and fast movements of all four limbs combined with lateral and vertical shifts of the trunk and pelvis, with challenging and progressive exercise programme, is appropriate to treat these disabling axial motor signs. This is in line with previous studies performed in PD patients that included highly challenging balance training programmes with significant improvements in balance control and reduction in falls rate [39], or virtual reality training on a treadmill with challenging gait speed or obstacle avoidance with significant increases in gait speed and stride length [13, 40]. The benefits were significant after at least 9 sessions of training. This reflects the need to perform at least a 40 min duration training session [26] and at least 8 sessions [9] to obtain clinically meaningful benefit.

The positive effects of our videogame on gait and balance performance in our PD patients could result from various physiological effects. This improvement could be related to increased corticospinal excitability of leg muscles as reported after Wii Fit training [41], thus leading to increased muscle strength [42], in particular for ankle movements with increased propulsive forces, as reflected by increased anteroposterior APAs displacement, step length and velocity, but less for hip movements with no significant change in mediolateral APAs displacement and step width [29, 38]. Our videogame could have induced motor learning in relation to the high attentional demand induced by the need to collect the maximum of coins, negotiate obstacles, while at the same time following an external, rhythmic and progressively increasing, auditory cue [12, 23, 43]. The fact that game performance significantly increased from session to session whereas game difficulty increased (with higher visual flow velocity of the virtual environment and number of movements to perform), and that the improvement of clinical gait and balance disorders (axial-UPDRS III subscore), gait speed and step length are persistent 3 months after the last session are in line with this hypothesis. The use of auditory and visual cues included in the game could also have promoted faster gait speed and longer step length as classically reported in PD patients [43, 44]. Rehabilitation was performed using visual feedback (avatar movement) that could lead to neuronal plasticity as reported in sports training [45] and in balance training while learning a new skill [46]. Lastly, gaming has been shown to induce dopamine release in healthy subjects, and thus could have induced parkinsonian disability improvement [47]. However, the absence of concomitant parkinsonian motor sign improvement fails to support this hypothesis.

The positive effects on freezing of gait, balance confidence and falls observed after 18 training sessions were not maintained 3 months after the intervention. Previous studies on physiotherapy in PD patients also report a loss of benefit within weeks after discontinuation of training [8]. However, recent data suggest that the addition of virtual reality component to physical training would yield longer-term benefit [13]. The fact that PD patients included in our study had a severe and advanced form of disease could have also negatively impacted the retention effects with reduced benefit of exercise after cessation of training, as also reported in previous studies [9]. Finally, these data confirm the importance of maintaining rehabilitation over time to obtain sustained benefit.

## Conclusions

These preliminary data suggest that rehabilitation based on a customised videogame to treat gait and balance disorders of PD patients is feasible, well accepted, and may be effective. The fact that the benefits of videogame training dissipate over time, as reported in other physiotherapy trials in PD, confirms the need of sustained exercise training in PD [48]. Further larger and controlled studies are needed to confirm these data, to examine the usability by the patients themselves of such cost effective rehabilitation strategies for training at home [20] and over longer periods, but also to investigate the retention effect and the underlying processes that contribute to motor improvement.

## Abbreviations

ABC: Activity Balance Confidence
APAs: Anticipatory postural adjustments
CoM: centre of mass, FOG = freezing of gait,
CoP: centre of foot pressure
FOG-Q: freezing of gait questionnaire
GABS: gait and balance scale
PD: Parkinson’s disease
PDQ-39: Parkinson’s Disease Questionnaire
UPDRS: Unified Parkinson’s disease rating scale
